# Characterization of HCV Interactions with Toll-Like Receptors and RIG-I in Liver Cells

**DOI:** 10.1371/journal.pone.0021186

**Published:** 2011-06-17

**Authors:** Erika A. Eksioglu, Haizhen Zhu, Lilly Bayouth, Jennifer Bess, Hong-yan Liu, David R. Nelson, Chen Liu

**Affiliations:** 1 Department of Pathology, Immunology and Laboratory Medicine, University of Florida College of Medicine, Gainesville, Florida, United States of America; 2 State Key Laboratory of Chemo/Biosensing and Chemometrics, School of Biology and Research Center of Cancer Prevention and Treatment of Hunan University & Hunan Tumor Hospital, Changsha, Hunan Province, China; 3 Department of Medicine, Division of Hepatobiliary Diseases, University of Florida College of Medicine, Gainesville, Florida, United States of America; Institut National de la Santé et de la Recherche Médicale, France

## Abstract

**Background and Aim:**

The aim of this study was to examine the mechanisms of IFN induction and viral escape. In order to accomplish the goal we compared our new hepatoma cell line LH86, which has intact TLR3 and RIG-I expression and responds to HCV by inducing IFN, with Huh7.5 cells which lack those features.

**Methods:**

The initial interaction of LH86 cells, Huh7.5 cells or their transfected counter parts (LH86 siRIG-I, siTLR3 or siTLR7 and Huh7.5 RIG-I, TLR3 or TLR7) after infection with HCV (strain JFH-1) was studied by measuring the expression levels of IFNβ, TRAIL, DR4, DR5 and their correlation to viral replication.

**Results:**

HCV replicating RNA induces IFN in LH86 cells. The IFN induction system is functional in LH86, and the expression of the RIG-I and TLR3 in LH86 is comparable to the primary hepatocytes. Both proteins appear to play important roles in suppression of viral replication. We found that innate immunity against HCV is associated with the induction of apoptosis by RIG-I through the TRAIL pathway and the establishment of an antiviral state by TLR3. HCV envelope proteins interfere with the expression of TLR3 and RIG-I.

**Conclusion:**

These findings correlate with the lower expression level of PRRs in HCV chronic patients and highlight the importance of the PRRs in the initial interaction of the virus and its host cells. This work represents a novel mechanism of viral pathogenesis for HCV and demonstrates the role of PRRs in viral infection.

## Introduction

Hepatitis C virus (HCV), a member of the family *flaviviridae*, infects approximately 170 million people worldwide leading to chronic liver disease in up to 80% of them [1], [2]. Failure to induce type I IFN, has been correlated with the lack of an appropriate innate response [3], [4]. In particular, chronically infected hepatocytes have a reduced expression of the pathogen recognition receptors (PRRs) and levels of interferon α (IFNα) which correlate with poor responses to standard treatment of care (Ribavirin and pegylated IFN) [5], [6]. How this down regulation occurs has not yet been fully elucidated.

An important mechanism of initial immune response is through the recognition of the virus' genetic material by PRRs, such as Toll-like receptors (TLR3 and TLR7 for RNA) and the cytosolic receptor DexH(D) RNA helicase retinoic acid inducible gene-I (RIG-I), followed by the induction of cytokines including type I IFN [7], [8], [9], [10]. This cytokine cascade starts with the binding of foreign RNA to specific sites inside the receptor followed by the coupling to adaptor molecules specific for each receptor (TRIF and Cardif respectively) that converge at transcription factors (IRF-3, NF-κB and ATF2/c-jun) in charge of genes related to the amplification of the signal and initiation of adaptive responses [9], [11], [12]. Both TLR3 and RIG-I share most of these pathways' molecules and can in theory have complementary effects on most cells. While these pathways are normally associated with the induction of cytokines it was recently discovered that RIP-1, a molecule normally associated with apoptosis by functioning downstream of TNFR by TRADD interaction, is also associated with TRIF or Cardif. This binding helps modulate the type of response to either pro-inflammatory or pro-apoptotic effects in an IRF-3-dependent manner [13], [14], [15]. Therefore, as long as both pathways are active (before pathogenic disruption) type I IFNs, pro-inflammatory cytokines and apoptosis can work together to induce clearance.

In this report, we demonstrate that HCV can induce IFNβ in the acute phase of infection, and that TLRs and RIG-I are involved in this process. Furthermore, we demonstrate that HCV viral envelope proteins can have an effect on this system by down regulation of these PRRs *in vitro*. This work demonstrates an indirect link between the actions of PRRs in viral infection which represents a novel mechanism of viral pathogenesis for HCV.

## Materials and Methods

### Cells culture, reagents and plasmids

LH86 cells have been developed by our group from a resected, well-differentiated, hepatocellular carcinoma and Huh-7.5 cells were kindly provided by Dr. Charles M. Rice (Rockefeller University, New York, NY)[16], [17]. All cell lines were propagated in DMEM supplemented with 10% FBS, 200 µM L-glutamine, non-essential amino acids, penicillin, and streptomycin (complete DMEM or cDMEM). The expression vector pTOPO was from Invitrogen (Carlsbad, CA) and the negative control siRNA was produced by T7 polymerase *in vitro* with the Ambion Silencer siRNA construction Kit or purchased (both control and kit from Ambion, Austin, TX). The plasmids for TLR3 and TLR7 (pUNO-hTLR3-HA and pUNO-hTLR7-HA) were purchased from Invivogen (San Diego, CA) and the siRNAs targeting those genes were purchased from Santa Cruz Biotechnology (Santa Cruz, CA). Core, Envelope E1/E2 and NS3/4A were amplified from pJFH-1 plasmid and cloned using TOPO TA Cloning kit from Invitrogen (Table 1 shows PCR primers used for amplification).

**Table 1:** Primer sets used for cloning.

	5′-TTGGATCCATGAGCACAAATCCTAAACC-3′
Core	5′-CGTCTAGATCAAGCAGAGACCGGAACGGTGAT-3′
	5′-GCTCTAGAGCCCAGGTGAAGAATACCAG-3′
E1/E2	5′-CGGGATCCTCATGCTTCGGCCTGGCCCAACAAG-3′
	5′-TTGGATCCATGGCTCCCATCACTGCTTATGCC -3′
NS3/4A	5′-CGTCTAGATCAGCATTCCTCCATCTCATCAAAAGCC-3′

Cells were transfected using Lipofectamine following the manufacturer's recommendations (Invitrogen). Briefly, in a 6-well tissue culture plate (Fisherbrand), 1×10^5^ Huh7.5 or LH86 cells were seeded in 2 mL of cDMEM and incubated at 37°C overnight. The next day 2 µg of DNA diluted in a total of 20 µL serum free media and 20 µL of lipofectamine diluted to a 100 µL were incubated for 45 minutes at room temperature before mixing. The mixture was incubated for another 15 minutes under the same conditions. After this incubation the cells were mixed with serum free media to a total 1 ml volume and layered on top of prewashed adherent cells. The transfected cells were incubated for another 24 hours before changing into complete DMEM. Stable cell clones were selected using antibiotics for a minimum of 4 weeks. All experiments were observed daily by light microscopy and cells collected for total RNA isolation with Trizol reagent (Invitrogen, Carlsbad, CA). Poly (I:C) was obtained from InvivoGen (San Diego, CA).

### HCV constructs and viral particle generation

pJFH-1 plasmid and pJFH-1/GND plasmid (negative control) were gifts from Dr. Takaji Wakita (Department of Virology II, National Institute of Infectious Diseases, Tokyo, Japan) [18]. The linearized DNA was purified and used as a template for *in vitro* transcription using MEGAscript kit (Ambion, Austin, TX). *In vitro* transcribed genomic JFH-1 or JFH-1/GND RNA was delivered into Huh-7.5 cells by electroporation. The transfected cells were transferred to complete DMEM medium and cultured for the indicated period. Cells were passaged every 3–5 days and corresponding supernatants were collected and filtered with a 0.45 µm filter device before freezing at −80°C. Viral titers were expressed as focus-forming units per milliliter (ffu/mL) and determined by the average number of NS5A-positive foci detected at the highest dilution of a serial dilution culture using Huh-7.5 cell line as host cells.

### Reverse Transcription and Polymerase Chain Reaction (RT-PCR)

RT-PCR of total RNA to obtain cDNA was performed using the Superscript II (50 U reverse transcriptase per reaction) first-strand synthesis for RT-PCR kit (Invitrogen) primed with oligo (dT)_12–18_ (Invitrogen) according to the manufacturer's instructions. After reverse transcription, cDNA was used for quantitative real-time RT-PCR using fluorophore-labeled LUX primers from Invitrogen, or SYBR (Table 2) some of which we have published before [19], [20], [21]. Reactions were conducted in a 96-well spectrofluorometric thermal cycler (StepOne Plus Sequence detector system, Applied Biosystems). Fluorescence was monitored during every PCR cycle at the annealing step. Results were analyzed with StepOne Plus software version 2.1 from Applied Biosystems. The PCR conditions were as follows: 50°C, 2 min; 95°C, 2 min (Super UDG master mix, Invitrogen) or 10 min (SYBR Green Master Mix, Applied Biosystems) and 40 cycles of 95°C, 15 s; 60°C–62°C (depending on the primer set), 30 s and 95°C, 1 min. All genes were analyzed through the 2^-ΔΔCt^ method following previously described calculations [22].

**Table 2:** Primers sets used for Real time RT-PCR(D-Lux primers show fluorochrome in the sequence).

	5′-CCTTCTTTAATGGTGGCTCCAT-3′
HCV 3′ UTR	5′-GGCTCACGGACCTTTCACA-3′
	Probe: 5′-TTAGCCCTAGTCACGGCT-3′
	5′-CAGCCGATTCATCGAGCACTCGC[FAM]G-3′
IFNβ	5′-TTCCAGGACTGTCTTCAGATGG-3′
I-8U	5′CACGGTCATAGCATTCGCCTACTCCG[FAM]G-3′
	5′GTCACGTCGCCAACCATCTT-3′
	5′-GACTGCTCGGAGGAGGACTCGCAG[FAM[C-3′
G1P3	5′CAGGATCGCAGACCAGCTCA-3′
	5′-ATGGTACCTCATGGCTATGATGGAGGTC-3′
TRAIL	5′-AAGCGGCCGCTCATAGTGTATCATCCTGAAAACTGA-3′
	5′-GGGTCCACAAGACCTTCAAGT-3′
DR4	5′-TGGTGTAACCCACACCCTCT-3′
	5′-AGAGGGATTGTGTCCACCTG-3′
DR5	5′AATCACCGACCTTGACCATC-3′
	5′-CATCTGCCTCCCCATATTCCT-3′
RANTES (CCL5)	5′-GCGGGCAATGTAGGCAAA-3′
	5′-ATTATTCCTGCAAGCCAATTTTG-3′
IP-10 (CXCL10)	5′-TCACCCTTCTTTTTCATTGTAGCA-3′
	5′-ACAACTTAGCACGGCTCTGGA-3′
TLR3	5′-ACCTCAACTGGGATCTCGTCA-3′
	5′-GACTGGACGTGGCAAAACAA-3′
RIG-I	5′-TTGAATGCATCCAATATACACTTCTG-3′
	5′-CGACCGGAGTCAACGGATTTGGT[JOE]G-3′
GADPH	5′-GGCAACAATATCCAGTTTAGCA-3′

HCV copies were determined using a standard curve of JFH-1 full-length RNA transcribed *in vitro*. PCRs were run in duplicates and overall data represents triplicate experiments and are represented as means ±SEM. The Taqman FAM-labeled primer-probe set used in our experiments was originally designed for HCV genotype 1. Compared to genotype 2a there is a mismatch in the forward primer in the ninth nucleotide (5′ to 3′). However, this primer set was demonstrated in our laboratory to be highly efficient, even more so than our previously published 5′UTR primer when used with *in vitro* transcribed JFH-1. Furthermore, most of the experiments carried here were performed initially with both primers and similar results were obtained.

### Immunofluorescence

Cells were grown on glass coverslips later to be washed and fixed on ice with 5% acetic acid in 100% ethanol (Fisherbrand). Cells were washed with 1X PBS and incubated with either goat anti-human TLR3 (Santa Cruz Biotechnology), goat anti-human TLR7 (Santa Cruz Biotechnology), rabbit anti-human RIG-I (ProSci Inc, Poway, CA) or mouse anti-HCV NS5A monoclonal antibody (established at our institution) for 1 hour. The respective secondary antibody (donkey anti-goat IgG FITC conjugated for TLR3 and TLR7, goat anti-rabbit IgG FITC conjugated, goat anti-mouse IgG FITC) were purchased from Southernbiotech (Birmingham, AL). Nuclei were counterstained with DAPI (Vector Laboratories Inc, Burlingame, CA), followed by examination under a fluorescence microscope (Olympus).

### Western blot analysis

Cells were washed with PBS and lysed in RIPA buffer (150 mM sodium chloride, 1% Nonidet P-40, 0.5% sodium deoxycholate, 0.1% SDS, and 50 mM Tris-Cl [pH 8.0] supplemented with 2 µg/mL of aprotinin, 2 µg/mL of leupeptin, 40 µg/mL of phenylmethylsulfonyl fluoride, and 2 mM DTT). Twenty µg of protein was electrophoresed on a 10% SDS-polyacrylamide gel and transferred to a polyvinylidene difluoride membrane (Bio-Rad, Hercules CA). The membranes were then blocked overnight at 4°C in blocking buffer (PBS containing 0.1% Tween 20 (PBS-T) and 5% fat-free milk power) followed by probing with each specific primary antibody (see immunofluorescence section for primary antibodies and mouse anti-human β-actin from Santa Cruz Biotechnology) for one hour at room temperature. After washing thrice with PBS-T for 10 minutes each, the membrane was incubated with the appropriate HRP-conjugated secondary antibody (all from Santa Cruz Biotechnology), diluted in PBS-T, for 1 hour at room temperature and washed 3 more times. Proteins were visualized with Supersignal West Pico Chemiluminescent Substrate (Pierce Biotechnology, Inc, Rockford, IL).

### Flow Cytometry

Pelleted cells were incubated for 30 min at room temperature with antibodies (as in immunofluorescence section), washed with staining buffer (PBS +2% BSA +0.1% Na azide), and then fixed with 2% paraformaldehyde and stored at 4°C. For intracellular staining the BDcytofix/cytoperm kit (BD biosciences) was used with the antibodies following the manufacturer's protocol. Cells were analyzed on a FACSCalibur flow cytometer (BD Biosciences, Heidelberg, Germany). Quantitation was done by the CellQuest software (BD Biosciences; version 3.2.1). Isotype-specific immunoglobulin controls were run for each fluorochrome. Thirty thousand cells were analyzed for each sample.

The Annexin V-FITC apoptosis was assayed as described previously (BD Pharmingen, San Diego, CA) [23]. A minimum of 30,000 events per sample were acquired on a FACSCalibur Flow Cytometer (BD Pharmingen, San Diego, CA) and subsequently analyzed with CellQuest software (BD Biosciences; version 3.2.1, San Diego, CA).

## Results

### HCV induction of IFN in LH86 cells depends on active viral replication

**Figure 1:**
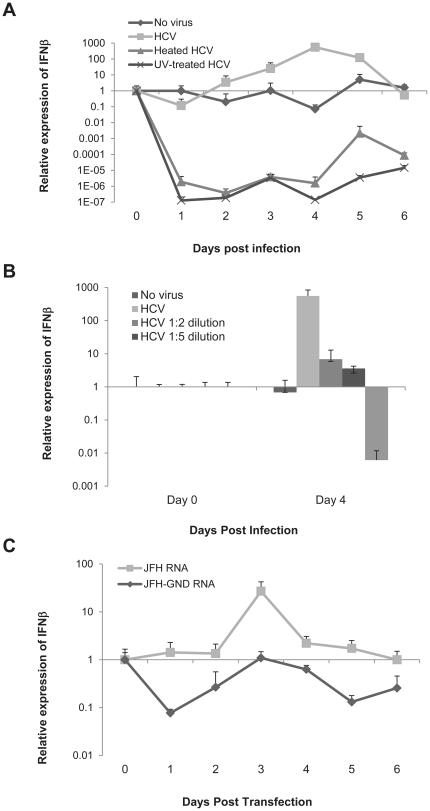
IFN response is dependent on viral replication. A) IFNβ mRNA expression was measured daily from the total RNA of LH86 cells treated with an MOI of 0.1 of HCV/JFH-1. The expression was calculated by the ΔΔCt method where uninfected cells were the experimental control and the housekeeping gene GAPDH was the internal control. Error bars represent the SEM of three separate experiments. The “No virus” control indicates cells that were cultured with uninfected Huh7.5 supernatant, “HCV” is the supernatant from infected Huh7.5 cells as described in the methods section (MOI = 0.1), “heated HCV” is the same supernatant as “HCV” but heated for 15 minutes at 72°C and “UV-treated HCV” was exposed to UV light for 15 minutes. B) IFNβ mRNA expression of different dilutions of virus (1∶1 MOI = 0.1) from day 0 and day 4 post infection calculated by the ΔΔCt method (determined as in part A). C) IFNβ mRNA expression of LH86 cells electroporated with *in vitro* transcribed HCV/JFH-1 or its non-replicating counterpart HCV/JFH-GND (day 0 is the day of the electroporation). Expression was calculated as described for part A.

We recently reported the induction of IFNβ by the hepatoma cell line LH86 in response to HCV [16]. Hence our first experiment was to corroborate that the IFN induced by LH86 cells leads to a functional response to the virus. We measured the expression of two interferon-stimulated-genes (ISGs) by Real time RT-PCR: 1-8U and G1P3 in these cells with or without viral infection by Real time RT-PCR. The experiment showed a fully functional IFN response with the production of both these ISGs. We then analyzed the expression of TLR3, TLR7 and RIG-I in LH86. Immunofluorescence and flow cytometry demonstrated a strong expression of RIG-I and TLR3 as well as a mild expression of TLR7 in these cells which was similar to primary hepatocytes, which were stained for comparison (data not shown). Therefore we used this system to examine if IFNβ expression correlates with functional HCV. We infected LH86 cells with HCV strain JFH-1 (genotype 2a) at a MOI of 0.1 or with the same amount of virus which was either heated at 72°C or exposed directly to UV light for 15 minutes. As seen in Figure 1A, induction of IFNβ expression correlated with intact virus, since inactivating the virus by heating or UV treatment down-regulated IFNβ expression.

In order to further understand the kinetics of this interaction we cultured LH86 cells with different dilutions of the virus. The data showed that IFN induction was titer-dependent (Figure 1B). To corroborate that viral replication, and not only RNA itself, is responsible for the induction of IFN, we electroporated LH86 cells with *in vitro* transcribed JFH-1 RNA or JFH-1/GND RNA. The JFH-1/GND virus has a mutation in its RdRp which prevents its replication but it is otherwise the same as JFH-1 [18]. We found that JFH-1, and not JFH-1/GND, induced IFNβ in LH86 cells indicating that there is a dependence on replication for its induction (Figure 1C). Interestingly, the basal levels of IFN after transfecting JFH-1/GND was comparable to LH86 cells by themselves (Compare Figure 1C with 1A) and we observed no down-regulation as we did with full virus (genetic material plus virion).

### Both TLRs and RIG-I play a role in the induction of IFN by HCV

**Figure 2:**
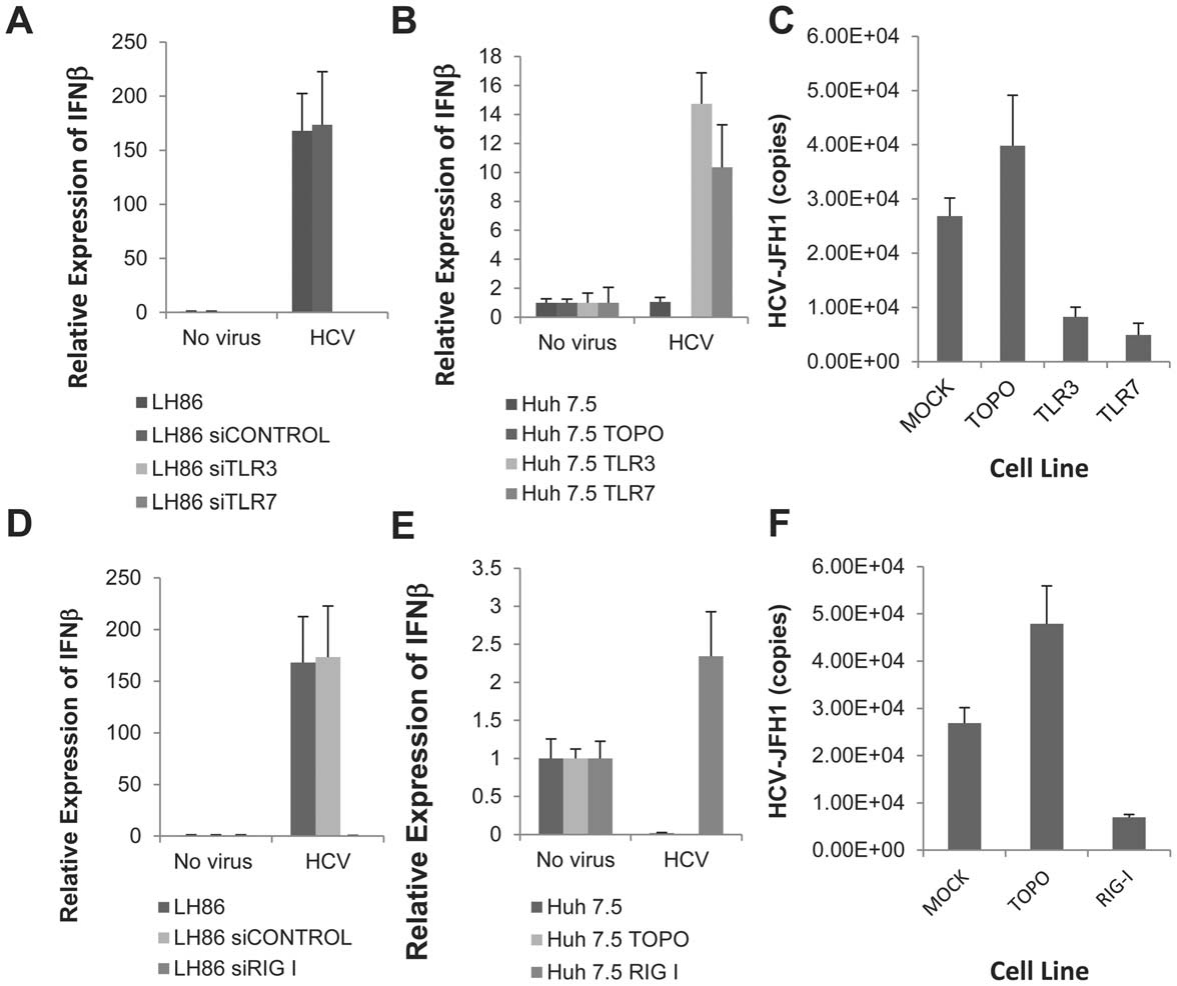
Both TLRs and RIG-I prevent viral replication and are needed for the induction of IFN. A) IFNβ mRNA expression at day 4 post-infection in LH86 cells after transfecting with a control siRNA, or siRNA against TLR3 or TLR7. Expression was calculated by the ΔΔCt method where uninfected cells were the experimental control and the housekeeping gene GAPDH was the internal control. Error bars represent the SEM of three separate experiments. No virus indicates cells were cultured with the same volume in uninfected Huh7. 5 supernatant, HCV is the supernatant from infected Huh7.5 cells as described in the methods section (MOI = 0.1). B) IFNβ mRNA expression at day 4 post-infection in Huh7.5 cells expressing TLR3 or TLR7. Expression was calculated by the ΔΔCt method as described in part A and bars represent the SEM of three separate experiments. C) Viral replication in Huh7.5 cells expressing TLR3 or TLR7 at day 7 after infection. HCV copy numbers were calculated by real time RT-PCR run with an HCV standard curve. D) IFNβ mRNA expression at day 4 post-infection in LH86 cells with silenced RIG-I. Methodology as described in part A. E) IFNβ mRNA expression at day 4 post-infection in Huh7.5 cells transfected with RIG-I. Methodology as described in part A. F) Viral replication in Huh7.5 cells transfected with RIG-I after 7 days of culture. Methodology as described in part C.

The role of TLRs and RIG-I in directly recognizing HCV or modulating its replication has not been fully characterized. We decided to do that by using a comparative cell culture system based on the distinct innate immune phenotype of two hepatoma cell lines: LH86 and Huh7.5. The first, LH86 cells, are a well-differentiated hepatoma cell line that has a fully functional set of PRRs and is capable of inducing IFN in response to HCV infection [16]. Conversely, Huh7.5 cells do not express TLR3 or TLR7, and have a RIG-I mutation, which may be a reason why these cells are so permissive to viral replication [3], [24], [25]. To test this hypothesis, we first silenced TLR3 and TLR7 in LH86 cells using specific siRNAs. As shown in Figure 2A, silencing these receptors abrogated the IFN response in LH86 cells. Conversely, transfecting TLR3 or TLR7 into Huh7.5 cells, followed by viral infection, rendered the cells capable of IFN induction (Figure 2B). Furthermore, forced expression of the same receptors into Huh7.5 cells significantly inhibited viral replication levels in these normally permissive cells (Figure 2C). Interestingly, the expression of RIG-I did not show the same phenomenon. While removal of RIG-I from LH86 cells significantly abrogated the IFNβ mRNA expression, delivering it into Huh7.5 cells did not increase IFN production to the same fold difference as LH86 cells or to the levels of Huh7.5 cells transfected with TLRs (150 versus 2 fold and 14 versus 2 fold respectively, Figures 2D and 2E) although it also considerably reduced the level of viral replication (Figure 2F).

### Cell death through RIG-I in response to HCV is linked to the TRAIL pathway

**Figure 3:**
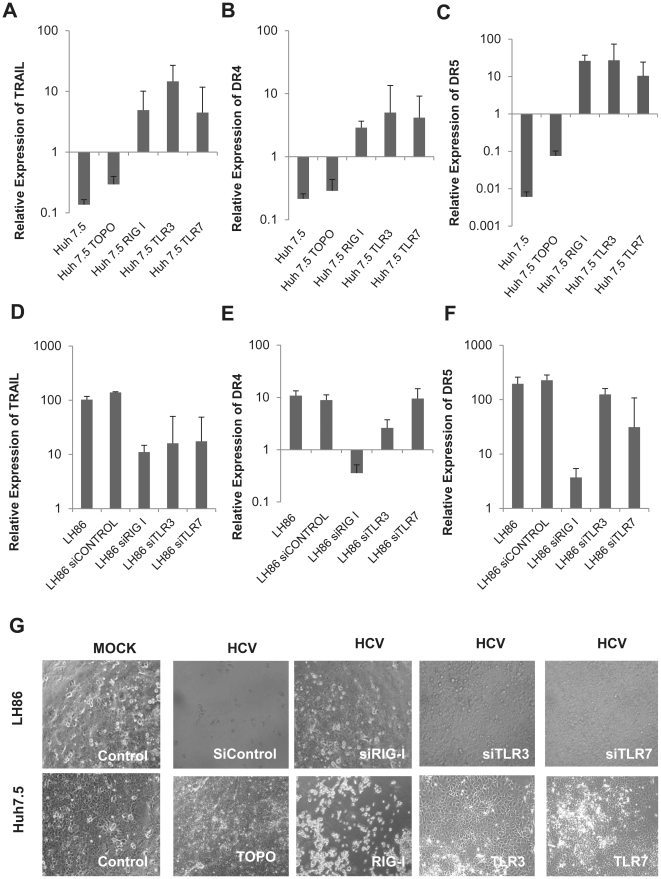
RIG-I is linked to the expression of TRAIL receptors DR4 and DR5. A) TRAIL mRNA expression 4 days after infection of Huh7.5 cells expressing RIG-I, TLR3 or TLR7. Expression was calculated by the ΔΔCt method where uninfected cells were the experimental control and the housekeeping gene GAPDH was the internal control. Error bars represent the SEM of three separate experiments. B) DR4 mRNA expression 4 days after infection of Huh7.5 cells expressing RIG-I, TLR3 or TLR7 following the methodology described in A. C) DR5 mRNA expression 4 days after infection of Huh7.5 cells expressing RIG-I, TLR3 or TLR7 following the methodology described in part A. D) TRAIL mRNA expression 4 days after infection of LH86 cells silenced for RIG-I, TLR3 or TLR7 calculated as described for part A. E) DR4 mRNA expression 4 days after infection of LH86 cells with silenced RIG-I, TLR3 or TLR7 calculated as described for part A. F) DR5 mRNA expression 4 days after infection of LH86 cells with silenced RIG-I, TLR3 or TLR7 calculated as described for part A. G) Phase view (100X magnification) of transfected cells 7 days after infection. Top row are LH86 cells and bottom row Huh7.5 cells. The labels note what each cell was transfected with.

Our previously published results showed that cytopathic effects in LH86 are related to the induction of TRAIL and its receptors DR4 and DR5, therefore we decided to investigate the relationship between the PRRs and the expression of these three genes [16]. As seen in figure 3, an interesting pattern appeared: forced expression of the PRRs in Huh7.5 cells increased the expression of the three genes studied (TRAIL, DR4, and DR5 in figures 3A, 3B and 3C respectively), but only silencing RIG-I, and not TLRs, had a profound effect on the expression of DR4 and DR5 after HCV infection (Figure 3D, 3E and 3F). Furthermore, RIG-I transfected Huh7.5 cells had visibly increased cell death comparable to the susceptibility of LH86 cells to HCV infection (Figure 3G).

### TLR3 and TLR7 suppress HCV RNA replication in hepatoma cells

**Figure 4:**
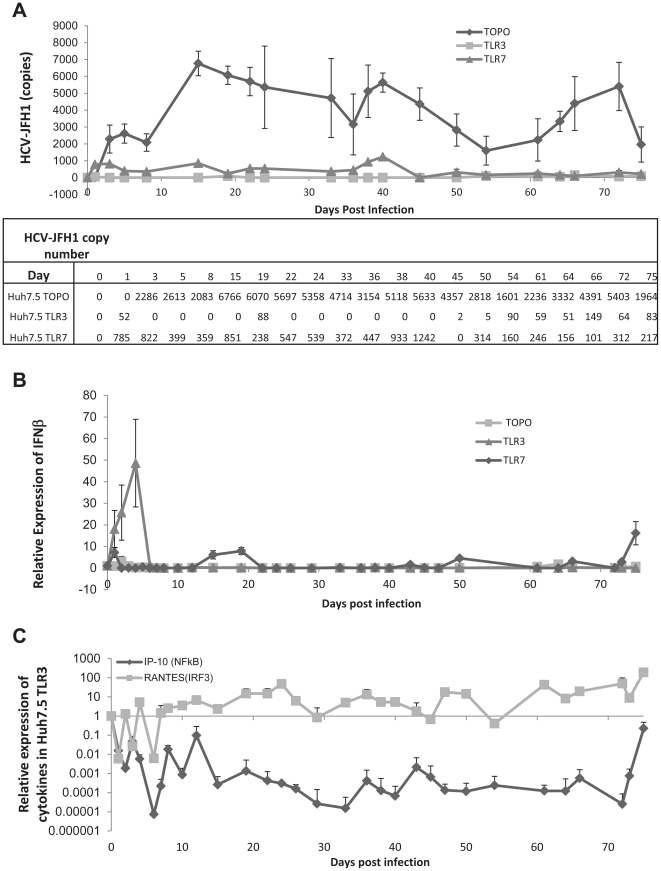
A strong initial IFN response is induced an IRF-3 response by TLR3 but is not enough to clear HCV. A) Viral replication in Huh7.5 cells stably transfected with TOPO (control) TLR3 or TLR7 infected with HCV MOI of 0.1 and collected every 2–3 days for RNA isolation (total 75 days). The HCV copy numbers from each time points were calculated by real time RT-PCR and compared against an HCV standard curve. B) IFNβ mRNA expression of the experiment described in part A. Expression was calculated by the ΔΔCt method where uninfected cells were the experimental control and the housekeeping gene GAPDH was the internal control. Error bars represent the SEM of three separate experiments. C) IP-10 and RANTES mRNA expression of the experiment described in part A. Methodology as described for part B.

To determine the functional role of TLR3 and TLR7, we examined the relationship between viral replication and the IFN response in a long-term culture system. The cells were infected with HCV-JFH1 virus and samples were collected every 2–3 days up to 75 days. Viral RNA replication levels were determined by real-time RT-PCR analysis. As shown in Figure 4, both TLR3 and TLR7 had a profound effect on viral replication throughout the time of analysis. The cells with both TLR3 and TLR7 suppressed HCV replication, while TLR3 has the most profound effect (Figure 4A). To test whether this antiviral effect correlates with an IFN response, we quantified IFNβ expression by real time RT-PCR. As shown in Figure 4B, over-expressing TLR3 in Huh7.5 cells induced a strong IFN production only in the first week, while TLR7 induced a very low level of IFN. TLR3 has at least two potential signaling pathways: NF-κB or IRF-3 cascades. To determine which pathway is operative under these conditions, we measured the expression of cytokines specific for each pathway (RANTES which is IRF-3 induced and IP-10 which is NF-κB induced) [26], [27], [28]. We observed that IP-10 was down-regulated in Huh7.5 TLR3 cells throughout the culture while RANTES was induced and sustained throughout the culture period indicating that the down regulation of viral replication correlates with the induction of the IRF-3 pathway without apparent NF-κB activation (Figure 4C).

### HCV can down-regulate the expression of TLR3 and RIG-I in LH86 cells

**Figure 5:**
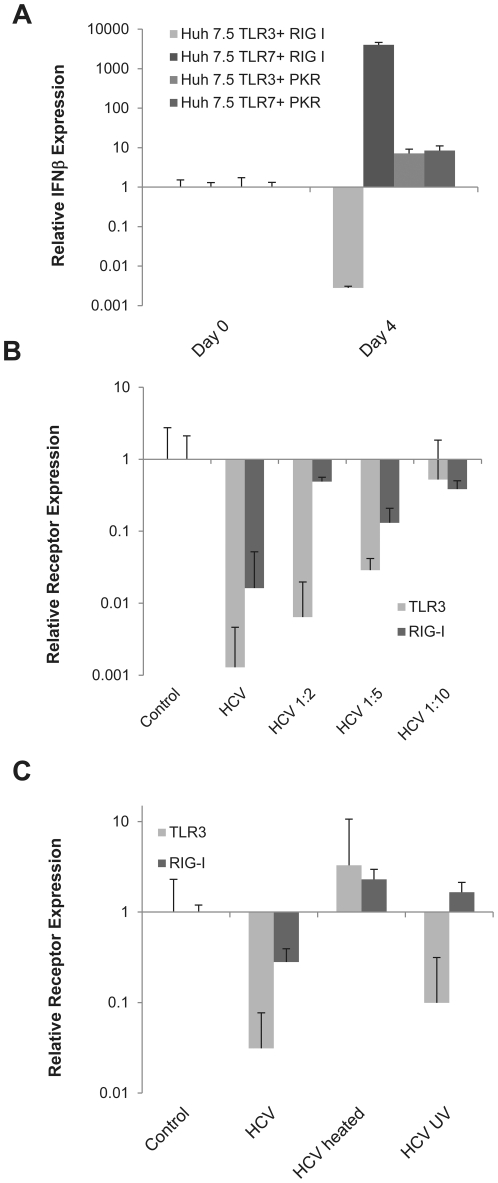
The expression of TLR3 and RIG-I is affected by intact virion proteins in hepatocytes A) IFNβ mRNA expression in Huh7.5 TLR3 or TLR7 stable cell lines co-transfected with either PKR or RIG-I after infection with HCV MOI = 0.1. Expression was calculated by the ΔΔCt method where uninfected cells were the experimental control and the housekeeping gene GAPDH was the internal control. Error bars represent the SEM of three separate experiments. B) TLR3 and RIG-I mRNA expression levels 7 days after infection with different HCV dilutions (methodology as in figure 1C). C) TLR3 and RIG-I gene expression levels 7 days after infection with normal, heated or UV-treated virus calculated as described in part A.

Since TLR3 and RIG-I have different functions, we hypothesized that their combined activation could lead to viral clearance. To test this hypothesis, we co-transfected the Huh7.5 TLR3 and TLR7 stable cell lines with RIG-I. We also did co-transfections of TLR3 and TLR7 cells with PKR, a different cytosolic receptor. While TLR7 did synergize with RIG-I to induce a strong IFN response, the TLR3/RIG-I combination did not show this effect. In these cells, contrary to our hypothesis, HCV infection down regulated IFN (Figure 5A). Furthermore, these effects are specific to TLR/RIG-I co-expressions in HCV infection since co-transfecting with PKR did not synergize or disrupt the IFN normally induced by either cell.

It has been reported that NS3/4A modulates TLR and RIG-I pathways at the level of the adaptor proteins TRIF and IPS-1 [24], [29]. Interestingly, studies have also demonstrated that expression of TLR3 and RIG-I are also reduced in HCV patients, therefore we decided to examine if there is a correlation between the expression of these receptors and virus infection. We measured the levels of both PRRs after infection with different dilutions of the virus. As shown in Figure 5B, HCV was able to reduce the level of TLR3 and RIG-I in a titer dependent manner. Furthermore, the virus proteins needed to be intact for TLR3 down regulation, since heating the virus prevented this down-regulation; similarly, RIG-I down regulation required intact virus but it also needed replicating virus since UV-inactivation abrogated the down-regulation of RIG-I, but not TLR3 (Figure 5C).

### HCV envelope proteins down regulate the expression of TLR3 and RIG-I

**Figure 6:**
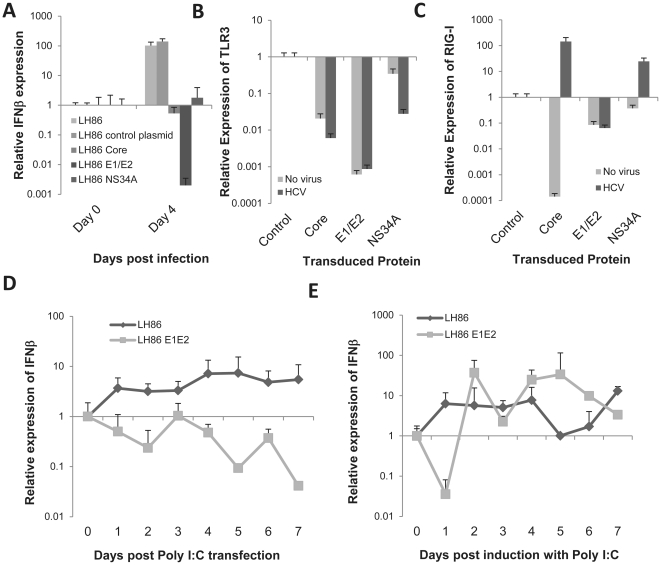
Envelope proteins affect the response to non-HCV responses through RIG-I receptor but only temporarily through TLR3. A) IFNβ gene expression of stably transfected LH86 cells expressing Core, E1E2 or NS3/4A 4 days after HCV infection. Expression was calculated by the ΔΔCt method where uninfected cells were the experimental control and the housekeeping gene GAPDH was the internal control. Error bars represent the SEM of three separate experiments. B) TLR3 mRNA expression 7 days after infection of stably transfected LH86 cells carrying the HCV proteins Core, E1/E2 or NS3/4A. Expression determined as described in part A. E) RIG-I mRNA expression 7 days after infection of stably transfected LH86 cells carrying HCV proteins Core, E1/E2 or NS3/4A. Expression determined as described in part A. D) IFNβ mRNA expression of LH86 cells or LH86 stably transfected with E1/E2 treated with 50 µg/µL transfected Poly I:C. Expression was calculated as described in part A. E) IFNβ mRNA expression of LH86 cells or LH86 stably transfected with E1/E2 treated with 50 µg/µL extracellular Poly I:C. Expression determined as described in part A.

The observations just described combined with our initial evidence (Figure 1A) demonstrates that non-replicating virus can down-regulate IFN which indicates there is likely a role by virion proteins in immune-evasion. To understand how these HCV proteins can impact the innate immune system we created stable cell lines with LH86 expressing three viral proteins: core, envelope (E1/E2) and NS3/4A (already known to affect IFN expression) [30]. These stable cell lines were infected with HCV at an MOI of 0.1 as before and the IFN response was measured. As expected, LH86 cells transfected with NS3/4A had a reduced IFN peak at day 4 as compared to control cells (Figure 6A). In contrast, LH86 cells transfected with core or envelope did not induce IFN. Core-transfected cells did have an earlier peak (not shown) but by day 4 IFN was no different than uninfected cells. Conversely, LH86 cells that harbored the envelope proteins did not express IFN at any time point during a 7 day culture and at day 4 the basal IFN expression on those cells was down regulated as well. Furthermore, these transfected cells were able to down-regulate the levels of TLR3 (Figure 6B) and RIG-I (Figure 6C) but only the envelope-transfected cells maintained the down-regulation of RIG-I after HCV infection.

Our data indicates that virion proteins are involved in evasion through the down-regulation of the expression of TLR3 and RIG-I. We also show that envelope proteins have bigger role in this evasion than other viral proteins tested. To corroborate the role of envelope in affecting the function of these receptors examined the kinetics of IFN induction in LH86 cells, with or without transfected envelope proteins, after Poly I:C stimulation. It is known that this ligand stimulates both RIG-I (by transfection of poly I:C) and TLR3 (when added directly to the media). Both transfected poly I:C (Figure 6D) and extracellular poly I:C (Figure 6E) were able to induce IFN in the absence of E1E2 protein in these cells. However, E1E2 affected the IFN response in LH86 cells stimulated with extracellular poly I:C for one day, while it completely abrogated the IFN response to transfected poly I:C. These results show that E1E2 can interfere with the RIG-I pathway, but only modestly interfere with TLR3-mediated IFN induction pathway.

## Discussion

The establishment of new *in vitro* models for the study of HCV has introduced some of the current concepts of how it interacts with the immune response and provided insight into these mechanisms. In the present study we observed that initial HCV's replication correlates strongly with the production of IFNβ in hepatocytes and that these responses are linked to the recognition of HCV by TLR3, and RIG-I. All these molecules have been strongly implicated as targets of viral evasion [24], [29], [31]. Previous studies have demonstrated that both TLR3 and RIG-I pathways are direct targets of NS3/4A serine protease of HCV. Furthermore, both receptors are: active in normal liver cells, known to induce type I IFN, share many of the same pathways and are capable of up-regulating each other [3], [32], [33], [34], [35], [36]. Studies have shown that either TLR3 or RIG-I is required or that both produce the same effect [33], [37]. Our data shows that different stimuli can induce distinct effects. While studying their mechanisms we demonstrated that TLR3 and RIG-I have specific roles during HCV infection: TLR3 was strongly involved in the induction of IFNs and creating an IRF-3 dependent environment that helps keep the viral replication low. RIG-I, on the other hand, can also involve the induction of cellular apoptosis through the TRAIL pathway and the death receptors DR4 and DR5. In addition, we also uncovered a potential mechanism of evasion by the viral envelope protein. Our data shows an inverse correlation on the presence of viral envelope proteins in hepatocytes with a decrease in the mRNA expression of both TLR3 and RIG-I. Furthermore, the stable expression of the envelope protein prevented the stimulation of RIG-I by poly (I:C) in the cytosol.

We showed a direct correlation between viral titers and levels of IFNβ suggesting that a threshold needs to be reached before an appropriate response is developed. This corroborates previously published data that also shows a delayed IFN response in HCV infection [34]. They also showed that TLR3's role in HCV was to induce IFN, IRF-3 activation and ISG induction in HCV-infected liver cells both *in vitro* and in clinical samples which correlates with our findings [3], [34]. Furthermore, our data suggests that the amount of IFN induced by HCV depends on the expression levels of TLR3 itself at the time of infection.

Based on our study, it is likely that the combination of both receptors should be optimal for the clearance of HCV infection. Our data demonstrates that RIG-I's main role may lie in the induction of apoptosis a function which was recently demonstrated for helicases [37], [38], [39], [40], [41]. We linked the induction of TRAIL-induced apoptosis in HCV to RIG-I and in particular to DR4 and DR5. This effect is usually attributed in other infections to TLR3 but although both TLR and RIG-I were able to up-regulate TRAIL, DR4 and DR5, when present, only the latter had a profound effect in the expression level of both death receptors. In general our findings can have strong implications in the study of the consequences of chronic infection since cytopathic effects have been implicated in the pathogenesis of HCV-induced liver injury in a TRAIL-dependent manner [42], [43], [44], [45]. Furthermore, clinically the levels of TRAIL are up regulated in chronic patients which correlate the availability of TRAIL with the induction of liver damage [46], [47].

**Figure 7:**
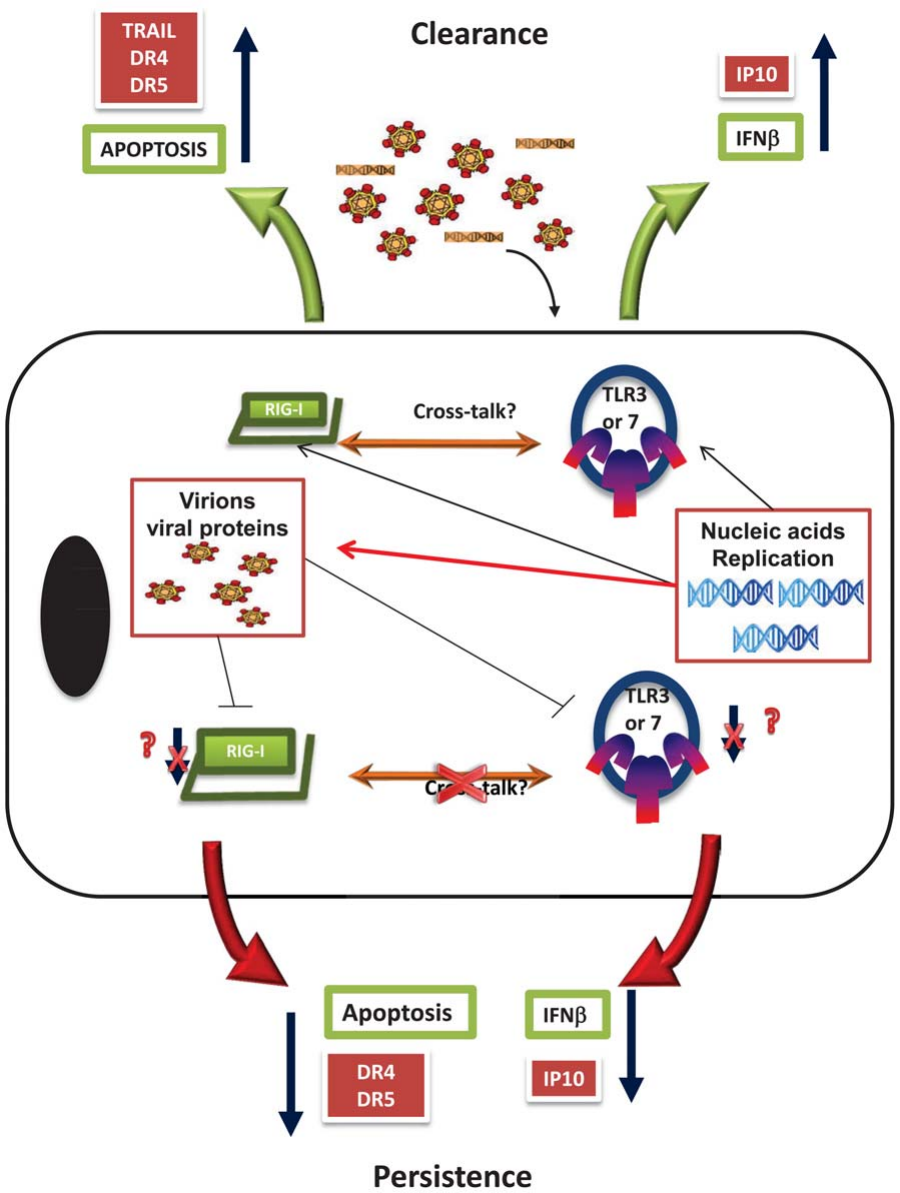
Schematics of the initial interaction of HCV in hepatocytes. Virus binds and gets in the cells were nucleic acid or replication induces the host cells innate immunity through the induction of apoptosis (mediated by RIG-I) and IFNβ (through TLR3 engagement). While the replication of the virus leads to the development of viral proteins like NS34A that interact downstream of these effects (red arrow line) there is a possible earlier evasion strategy performed by virion proteins that lead to the down regulation of RIG-I and TLR3 preventing apoptosis and the induction of IFNβ. Apoptosis is particularly prevented by down regulating TRAIL receptors, DR4 and DR5, and IFNβ by preventing NF-κB pathways such as the one that induces IP-10. Together all of these factors help the cell survive and the virus to persist in the host's hepatocytes.

Interestingly, the expression of both receptors in Huh7.5 cells disrupted the induction of IFNβ seen with either receptor alone. This observation argues that the virus might exert certain pressure when they are in combination since this combination of receptors has been demonstrated to induce IFN in the absence of HCV [33], [48]. In these studies, the authors observed no synergistic effect between the receptors which led them to argue that there was no direct interaction with each other. Our overall data supports the idea that these two receptors may interact with each other and that they have specific functions in HCV infection (Figure 7). This discrepancy made us investigate the role of HCV on the levels of these receptors. We observed that the expression levels of TLR3 and RIG-I were down regulated after infection in LH86 cells. Our finding correlates with clinical observation, which shows that the levels of these receptors in patients are down regulated [5]. In our system those effects were mediated by intact viral proteins, in particular the virion proteins: core and envelope. These results correlate well with our recent studies on healthy myeloid-derived dendritic cells where virus did not replicate but was able to induce phenotypical changes as well as modulate the cytokine secretion [49]. Similarly, a recent report on HCV *in vitro* studies demonstrated that viral infection can specifically down regulate another receptor, TLR7 and envelope proteins have been directly linked to the blocking of the TRAIL pathway which could be mechanistically explained by the down regulation of RIG-I [16], [50], [51], [52]. This further supports our theory of the initial interaction of the virus with its host being dependent on the amount of virus present either preventing or delaying immune responses (Figure 7). Further studies would reveal if this correlation is indeed an evasion strategy of HCV and what might be the mechanism for this phenomenon.

In summary, we have characterized the interaction of HCV with innate immune molecules, TLR3, TLR7, and RIG-I. We found that TLR3 and TLR7 play an important role in controlling viral infection through the IFN production, while RIG-I play a role in IFN induction as well as in the induction of hepatocyte apoptosis. We also found that the presence of viral E1E2 protein correlates with lower TLR3 and RIG-I expression, a possible mechanism of HCV evasion of innate immune response. Beyond IFN, future studies should aim at understanding the specific role of NF-κB and IRF-3 cascades, on why the virus seems to choose one pathway over the other, and how this might interfere with viral clearance.
